# The structural, electronic, and magnetic properties of substitutional transition metal doping in CrSi_2_N_4_ monolayer

**DOI:** 10.1038/s41598-025-30838-0

**Published:** 2025-12-18

**Authors:** Mohamed A. Abdelati, Mohamed M. Fadlallah

**Affiliations:** 1 Department of Physics, Faculty of Science, Benha University, Benha, 13518 Egypt; 2 National Institute of Laser Enhanced Sciences, Cairo University, ElGiza, Egypt

**Keywords:** 2D materials, Density functional theory, Doping, Electronic and magnetic properties, Bandgap, Chemistry, Materials science, Nanoscience and technology, Physics

## Abstract

After the synthesis of two-dimensional (2D) structures, Mo(W)Si_2_N_4_ (Hong et al., 2020; Science 369 670), proclaimed the dawn of a new family of 2D materials, including CrSi_2_N_4_ (CrSiN) semiconductor, which has a band gap of 0.49 eV and boasts remarkable properties. In this regard, we conducted density functional theory to investigate the structural, magnetic, and electronic properties of mono cation doped CrSiN (TM-CrSiN) with substitutional 3d/4d transition metal (TM) (at the Cr site). The bond lengths of TM-N increase as the atomic size of TM increases, and the formation/binding energy can be explained by the atomic size and electronegativity of TM as compared to the corresponding value of Cr. The doped structures with Ti/Zr, V/Nb, Mo, and Mn/Tc are nonmagnetic monolayers. In contrast, the other doped monolayers are magnetic. The doped monolayers with Ti/Zr and Mo are semiconductors with a band gap of 0.20/0.25 eV and 0.52 eV, respectively. It is noteworthy that the structures with Sc/Y, Fe, Co/Rh, Ni/Pd, and Zn/Cd dopants are half-metallic, which makes them suitable monolayers for spintronic applications. We find Cu- and Ag-CrSiN sheets behave as metallic structures and could be utilized in spin-filter devices.

**Supplementary Information:**

The online version contains supplementary material available at 10.1038/s41598-025-30838-0.

## Introduction

Two-dimensional (2D) structures, with their exceptional physical and chemical properties, have attracted significant interest in materials research. They could be used in a wide range of applications, for instance, for batteries^1,2^, sensors^3–7^, spintronics^8–10^, and transistors^11–13^. The extraordinary properties of 2D materials have surprised researchers and made them a priority for replacing silicon and germanium in transistor applications^14^.

Following the successful experimental exfoliation of monolayer graphene^15–17^, many 2D materials have been experimentally and theoretically studied, including BeN_4_^18,19^, BeO^20,21^, C_6_N_7_^22,23^, and AlSb^24,25^. In 2022, 2D Mo(W)Si_2_N_4_ was successfully synthesized via chemical vapor deposition^26^. The family of MA_2_Z_4_ (M = Ti, V, Cr, Hf, Zr, Nb, or Ta; A = Si or Ge; Z = N, P, or As) has been theoretically predicted^27,28^. Theoretical calculations demonstrated that MSi_2_N_4_ (M = Mo, W, or Cr) has the highest piezoelectric coefficient compared to other known 2D materials^28^. Furthermore, the theoretical results indicate that MX_2_Y_4_ nanosheets have the potential to compete with graphene in numerous applications, including nanoelectronics, optoelectronics, energy storage, conversion, and thermal management systems, outperforming not only the transition metal dichalcogenides group but also other 2D materials^28^.

The electronic and magnetic properties of MoSi_2_N_4_ were systematically modified through anion substitution, point defects, and molecular doping^29–31^. Doped Mo(W)Si_2_N_4_ structures incorporating 3 d, 4d^32,33^, and 5 d^34^ transition metals were investigated to assess their electronic and magnetic properties, as well as their potential for water splitting^35^. Both pristine and B doped MoSi2N4 monolayers were explored as sensors for environmental^36^ and hazardous gases^37^. Regarding energy storage applications, the adsorption of metal ions onto MA_2_Z_4_ monolayers demonstrated effective anchoring for lithium–sulfur batteries^38^. Furthermore, studies on alkali metal adsorption on MSi_2_N_4_ (M = Ti, V, Mo, W) indicate that these monolayers are promising candidates for low-cost metal-ion batteries^39–41^. Additionally, heteroatom doping of the MoSi_2_N_4_ structure was shown to enhance the adsorption of lithium and sodium, supporting their application in lithium and sodium batteries^42^. MA_2_Z_4_ monolayers were also used as catalysts for the O_2_ reduction reaction^43^. Furthermore, metal doped MoSi_2_N_4_ exhibited catalytic activity for O_2_^44^, CO^45^, and CO_2_^46^ reduction reactions, as well as for the hydrogen evolution reaction^47^.

The CrSi_2_N_4_ (CrSiN) monolayer is a semiconductor with a bandgap of 0.49 eV^28^. Many publications are concerned with the CrSiN monolayer, particularly for Na batteries^48^, where the rich Ti-CrSiN monolayers are noteworthy candidates for generating a spin current source^49^. CrSiN can redshift the ultraviolet-visible spectrum when compared to MoSi_2_N_4_^50^. Furthermore, achieving n- and p-type Ohmic contacts in vertical CrSiN/graphene^51^ and CrSiN/metal monolayers (Ti_2_C, NbS_2_, and Ti_3_C_2_) heterostructures^52^ is investigated. Additionally, it has been found that the strain can induce magnetism in CrSiN^53^. Furthermore, the CrSiN/Cs_2_SnI_6_ shows promise for applications in photoelectronic and photovoltaic devices^54^.

Our study provides a comprehensive analysis of transition metal doping in CrSiN sheets, motivated by their remarkable performance. To achieve this, we employ first principles calculations to systematically examine substitutional doping at the Cr site with transition metals from the 3 d and 4 d series. Specifically, we investigate the geometry, stability, electronic structure, and magnetism of these doped sheets. This analysis enhances understanding of 2D magnetic materials and offers new insight into the physical chemistry of transition metal doped CrSiN, paving the way for broad applications in nanoelectronic semiconductors and spintronic devices.

### Computational methods

All calculations were performed by the QUANTUM ESPRESSO plane-wave density functional theory package^55^. Spin-polarized computations were employed to determine the electronic and magnetic properties of the system. For the exchange-correlation functional, we used the generalized gradient approximation (GGA) in the scheme of Perdew–Burke–Ernzerhof (PBE). The electron–ion interactions were described using scalar-relativistic ultrasoft pseudopotentials^56^. The calculated band gap of Mo(W)Si_2_N_4_ using the PBE exchange correlation is closer to the experimental band gap than that of HSE06^32,33,57^. An 18 × 18 × 1 Monkhorst–Pack k-mesh and a 45 Ry energy cut-off were utilized. Our systems are structurally relaxed until the force on each atom is less than 0.001 Ry/Bohr. We constructed a 3 × 3 CrSiN supercell, with an 18 Å thick vacuum separation to prevent interactions along the c-direction. With 63 atoms, the doping concentration in the supercell is 1.6%. When examining the vdW interaction (DFT-D2) effect for many cases, the calculated electronic structures have not altered, which is consistent with the fact that the vdW interactions in monolayer structures can be disregarded^58–60^. The Löwdin charges are produced using projected Kohn–Sham wavefunctions onto localized atomic-like orbitals. Accordingly, the Löwdin charge indicates the amount of electron density that an atom has gained or lost to the surrounding atoms. The thermodynamic stability of pristine CrSiN and transition metal doped CrSiN (TM-CrSiN) systems was evaluated using ab initio molecular dynamics (AIMD) simulations at 400 K. Simulations were performed in a canonical (NVT) ensemble with a time step of 1 fs over a total duration of 5 ps.

## Results and discussion

### Structures

**Fig. 1:**
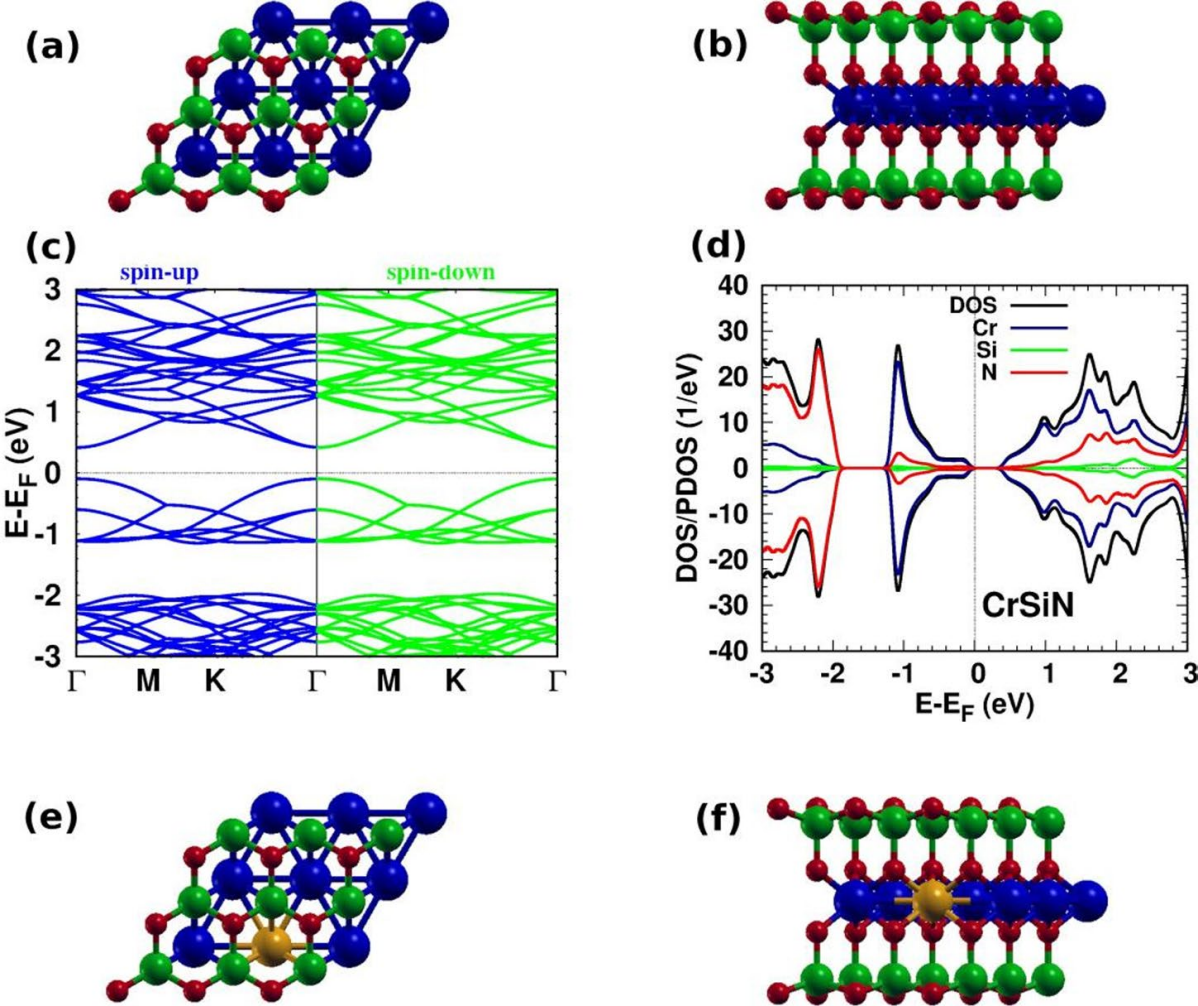
(**a**) Top and (**b**) side views of the pristine CrSiN monolayer, (**c**) band structure, and (**d**) DOS/PDOS for the pristine CrSiN monolayer. (**e**) Top and (**f**) side views of doped CrSiN monolayer (N (red), Si (green), Cr (blue), and TM dopant (dark yellow)).

**Fig. 2:**
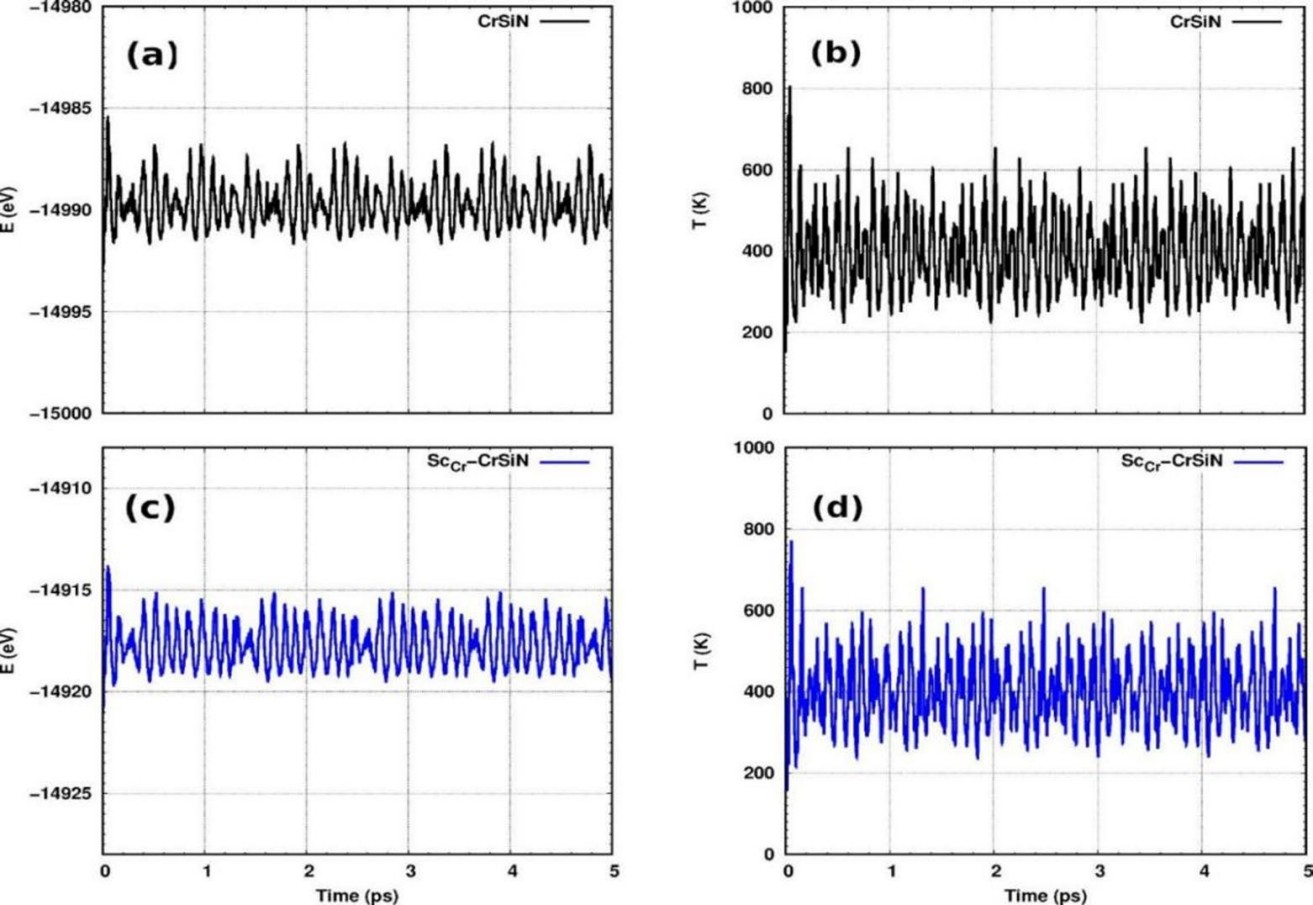
Total energy and temperature variations over 5 ps (10000 steps) during the AIMD simulation at 400 K for (**a**,** b**) CrSiN and (**c**,** d**) Sc-CrSiN.

Seven atoms make up each unit cell of the CrSiN monolayer, which has the order N-SiN-Cr-N-Si-N (Fig. 1(a)/(b)). A layered structure consists of the CrN_2_ layer sandwiched between two slightly buckled SiN layers. We find that the Si-N, Cr-N, and Cr-Cr bond lengths are 1.75 Å, 2.00 Å, and 2.84 Å, respectively, which are consistent with previous calculations^50^. During the simulations, fluctuations in energy and temperature appear. Despite these fluctuations, the pristine CrSiN and the doped structures remain stable, as demonstrated by the consistency of the average total energy and temperature variations (Fig. 2). This confirms that pristine CrSiN and the dopant structures are thermodynamically and dynamically stable. Furthermore, Figures S1–S5 in the supplementary material display molecular dynamics for the other doped systems.

**Fig. 3:**
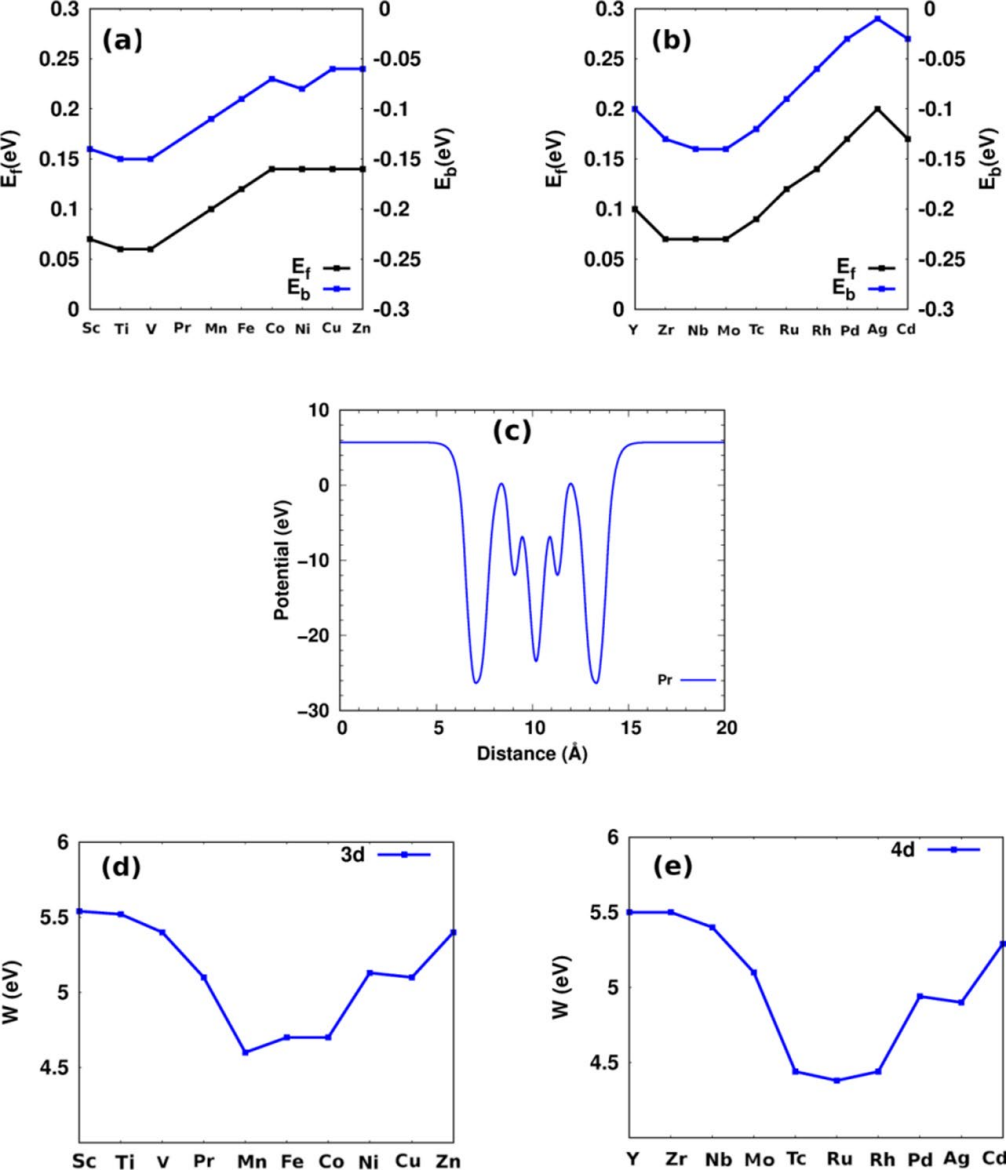
The formation (E_*f*_) and binding (E_*b*_) energy: (**a**) 3d- and (**b**) 4d-CrSiN. (**c**) The potential average of the pristine (Pr) CrSiN monolayer. The calculated work functions for (**d**) 3d- and (**e**) 4d-CrSiN.

The formation (E_*f*_) and binding (E_*b*_) energies are calculated using the following formulae to assess the stability of the metal doped CrSiN (TM-CrSiN):

1 $${E_f}={\text{ }}{E_{TM - CrSiN}}+{E_{Cr}} - ({E_{CrSiN}}_{+}{E_{TM}}),$$

2 $${E_b}={\text{ }}{E_{TM - CrSiN}} - {E_{TM}} - \left( {{E_{vac - CrSiN}}} \right),$$

where *E*_*TM−CrSiN*_ and *E*_*CrSiN*_ denote the energy of the metal doped and pristine CrSiN, respectively; *E*_*Cr*_ and *E*_*TM*_ are the total energy of Cr and the isolated metal dopant atom, respectively. *E*_*vac−CrSiN*_ is the energy of CrSiN with a single Cr vacancy. The behviour of the formation and binding energies shows the same trend, indicating the stability of doped structures (Fig. 3(a)/(b)). The most stable structure is Mn-CrSiN due to the similarity of the atomic radius of Mn and Cr (139 pm). As the difference in the atomic radius of Cr and TM increases, the formation energy and binding energies decrease.

The calculated formation energy and binding energy for CrSiN doped with 3 d and 4 d metals exhibit a similar periodic trend (Fig. 3(a)/(b)). The variance is mostly controlled by atomic size, which raises the formation energy and lowers the binding energy for dopants that precede Cr on the periodic table. However, the trend depends on both atomic size and electronegativity of the dopants following Cr. This shift in the regulating parameters reflects the relationship between structural strain (size effect) and electronic interaction (electronegativity effect) during the 3d/4d metals.

The work function is *W* = *E*_*vacuum*_ −*E*_F_, where *E*_*vacuum*_ and *E*_F_ stand for the vacuum and Fermi energies, respectively. The electrostatic potential of the pristine sheet is shown in Fig. 3(c), which demonstrates symmetry around the middle layer and flatness in the vacuum region. This behavior is consistent across all TM-CrSiN monolayers. Figure 3(d)/(e) illustrates how the work functions (W) for TM-CrSiN monolayers vary with 3 d and 4 d metals in the periodic table. For 3d/4d transition metals, the work functions range from 4.60/4.38 eV (Mn/Ru) to 5.50/5.54 eV (Sc/Y). Mn/Ru-CrSiN shows a higher probability of electrons escaping from its surfaces than the other doped structures. Table 1; Fig. 3(d)/(e) show that the work function trend is related to electronegativity. An increase in electronegativity decreases the work function. Notably, the work function for structures with TM = 4 d (from Mo to Cd) is lower than for structures with TM = 3 d (from Cr to Zn). This results from the significant electronegativity difference between 4 d and 3 d metals, such as from Mo to Pd. Consequently, 4d-CrSiN monolayers generally exhibit a higher probability of electron escape than 3d-CrSiN monolayers. Moreover, the work function decreases as the TM-N bond length decreases. As bond strength increases from Sc/Y to Mn/Tc, the work function also decreases. For other dopants, both the electronegativity and bond length of TM-N play important roles in determining the work function value^61^. The work function of pristine CrSiN (5.1 eV) is slightly smaller than that of pristine MoSi_2_N_4_ (5.2 eV) because the Cr-N bond length (2.0 Å) in pristine CrSiN is shorter than the Mo-N bond length (2.9 Å) in pristine MoSi_2_N_4_^32^.

The spin-resolved DOS at the Fermi energy (*E*_*F*_) is crucial for spintronic and spin-filter devices. As a result, the spin polarization ratio (SPR (*E*_*F*_)) is eventually determined at the Fermi level^62,63^.

3 $$\:SPR\left({E}_{F}\right)=\frac{S{D}_{\uparrow\:}\left({E}_{F}\right)-S{D}_{\downarrow\:}\left({E}_{F}\right)}{S{D}_{\uparrow\:}\left({E}_{F}\right)+S{D}_{\downarrow\:}\left({E}_{F}\right)}$$

Where SD_↑_ and SD_↓_ refer to DOS of the spin-up and spin-down states, respectively. The SPR values are all shown in Table 1. The SPR of doped CrSiN with Sc, Y, Fe, Co, Rh, Ni, Pd, Zn, and Cd is 100%, indicating that they can be used in spintronic applications. Additionally, doped CrSiN with Cu can be utilized for spin-filter applications.

The bond lengths of TM-Cr/N are presented in Table 1. The bond lengths TM-Cr decrease as the TM ionic size decreases, from Sc/Y to Mn/Tc. However, the bond lengths increase from Fe/Ru (2.86 Å) to Ni/Pd (2.90 Å). For Cu/Ag and Zn/Cd dopants, the bond lengths are nearly constant (2.88/2.90 Å). Similar behaviours are observed for the TM-N bond lengths, except for the Ni-N bond, which exhibits a different trend than the Ni-Cr bond.

**Table 1:** Bond lengths (TM-N (Å)), magnetic moments (*Mag* (*µ*_*B*_)), formation energy (*E*_f_ (eV)), binding energy (*E*_b_ (eV)), spin polarization ratio (*SPR*) %, and work function (W (eV)) of TM-CrSiN.

TM	Sc	Ti	V	Cr	Mn	Fe	Co	Ni	Cu	Zn
	(Y)	(Zr)	(Nb)	Mo	(Tc)	(Ru)	(Rh)	(Pd)	(Ag)	(Cd)
TM-N	2.13	2.05	2.01	2.00	1.99	1.99	2.00	1.92	2.10	2.14
	(2.20)	(2.15)	(2.08)	(2.05)	(2.05)	(2.05)	(2.07)	(2.24)	(2.20)	(2.24)
*Mag*	1.09	0.0	0.0	0.0	0.0	1.84	2.91	1.83	4.55	1.93
	(1.19)	(0.0)	(0.0)	(0.0)	(0.0)	(1.51)	(2.91)	(1.94)	(4.54)	(2.37)
*E*_*f*_	0.07	0.06	0.06	0.0	0.10	0.12	0.14	0.14	0.14	0.14
	(0.10)	(0.07)	(0.07)	(0.07)	(0.10)	(0.12)	(0.14)	(0.17)	(0.196)	(0.173)
*E*_*b*_	−0.14	−0.15	−0.15	0.0	0.11	−0.09	−0.07	−0.07	−0.06	−0.06
	(− 0.10)	(− 0.13)	(− 0.14)	(− 0.14)	(− 0.12)	(− 0.09)	(− 0.06)	(− 0.03)	(− 0.01)	(− 0.03)
*SPR*	100	0.0	0.0	0.0	0.0	100	100)	100	71	100
	(100)	(0.0)	(0.0)	(0.0)	(0.0)	(31)	(100)	(100)	(42)	(100)
*W*	5.54	5.52	5.40	5.10	4.60	4.70	4.70	5.13	5.10	5.40
	(5.50)	(5.50)	(5.40)	(5.10)	(4.44)	(4.38)	(4.44)	(4.94)	(4.90)	(5.29)

### Electronic structure

The band structure and density of states for CrSiN show that the monolayer is a semiconductor with a band gap of 0.49 eV (Fig. 1(c)/(d)), consistent with many earlier investigations [28, 48, 50, 52, 64]. The projected density of states (PDOS) of the pristine CrSiN monolayer reveals that the contribution from Cr states is significant around the Fermi level. In contrast, N states dominate in the valence band below − 1.8 eV. Since Si states do not significantly contribute to the shown DOS range, we do not display their contribution in the following analysis of the doped structures.

**Fig. 4:**
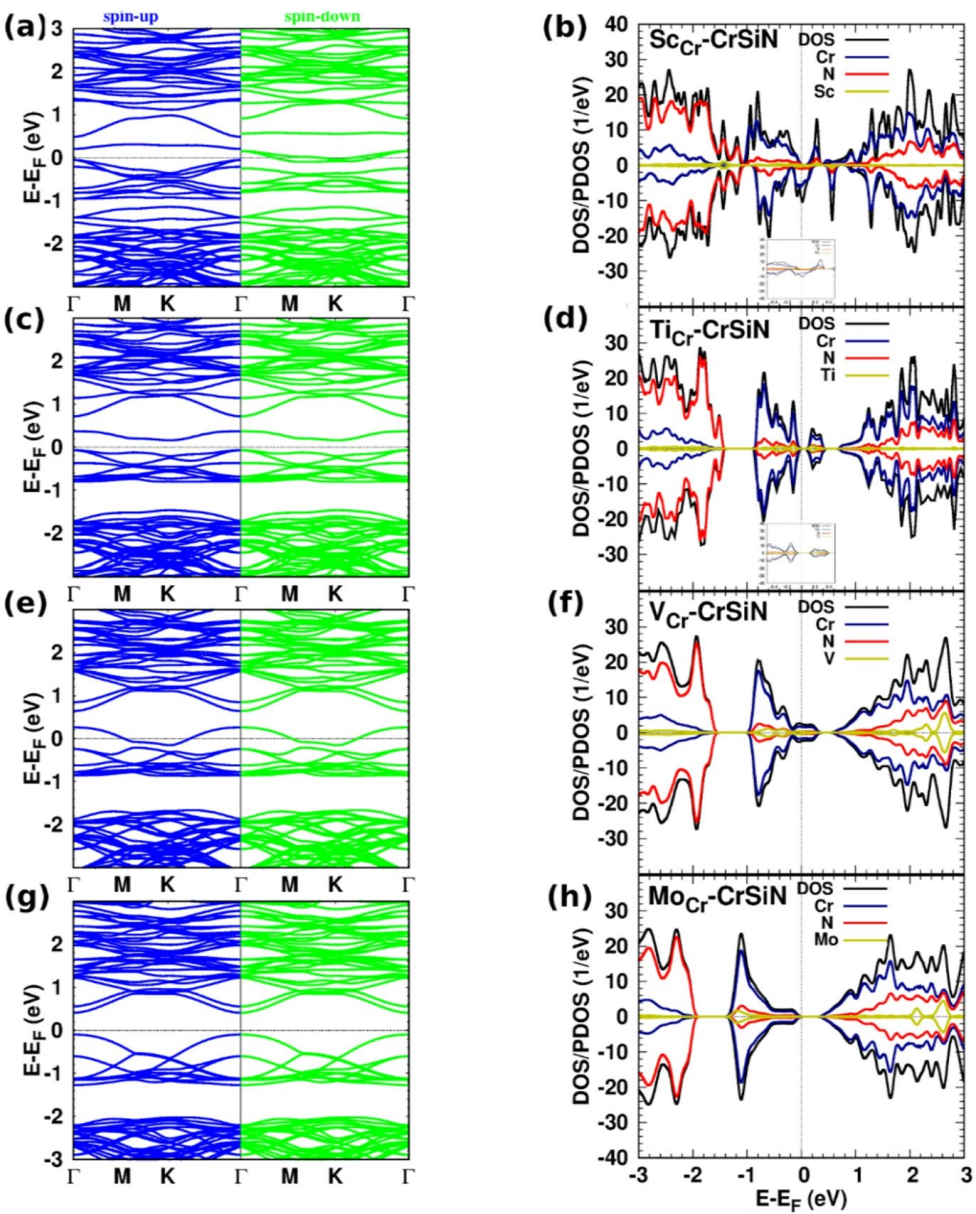
(The band structure, DOS/PDOS): (**a**,** b**) Sc-, (**c**,** d**) Ti-, (**e**,** f**) V-, and (**g**,** h**) Mo-CrSiN. The inset figures in panels (**b**) and (**d**) display the corresponding DOS/PDOS within a specified energy range.

Figure 4(a)/(b) shows the band structure and DOS of a monolayer of CrSiN with a substitutional doping with Sc doping. Compared to the DOS of the pristine monolayer, the entire DOS spectrum is distorted, with the contribution of Sc being insignificant. The Cr states dominate the energy range from − 1.0 eV to 3.0 eV, with a minor contribution from N states from − 1.0 eV to 1.0 eV. Furthermore, the N states dominate from − 1.2 to − 3.0 eV. The effect of Sc doping appears at the Fermi energy. The asymmetric behaviour between spin-up and spin-down DOS components is clear. The Sc-CrSiN is half-metallic, which may be employed in spintronic applications. The DOS/PDOS of Y-CrSiN is similar to the corresponding behaviour of Sc-CrSiN, particularly in the vicinity of the Fermi energy (see supplementary materials, Fig. S6).

**Fig. 5:**
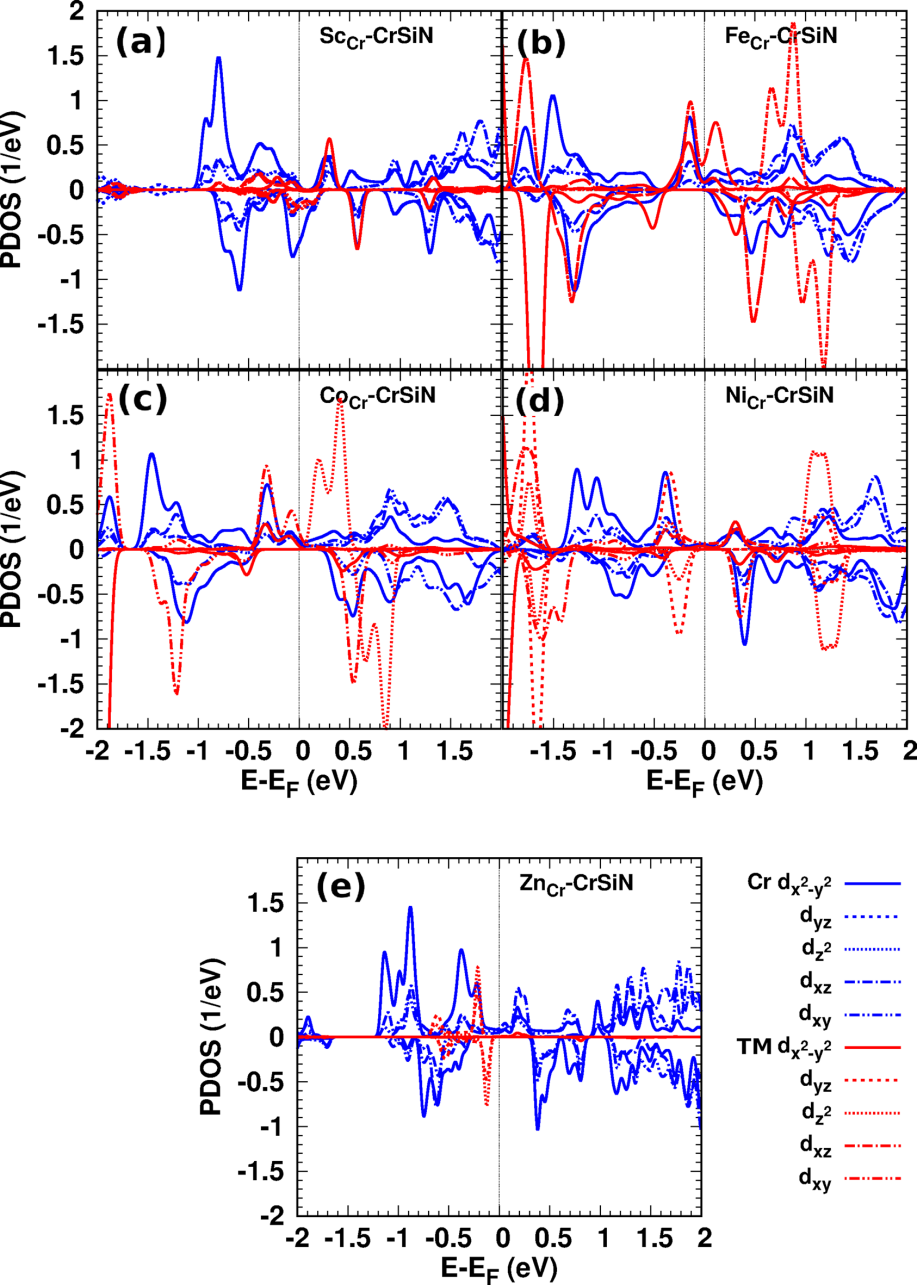
The PDOS for (3d) orbitals of Cr and TM: (**a**) Sc-, (**b**) Fe-, (**c**) Co-, (**d**) Ni-, and (**e**) Zn-CrSiN.

At the Fermi energy (spin-down), the dominant contribution to the states arises from the hybridization of Cr 3$\:{d}_{{x}^{2}-{y}^{2}}$ orbitals and significant contributions of 3($\:{d}_{xy},\:{d}_{xz})\:$orbitals from both Cr and Sc atoms (Fig. 5(a)). In Y-CrSiN, the main contribution comes from the hybridization between 3($\:{d}_{xy},\:{d}_{xz})\:$orbitals of Cr and 4($\:{d}_{xy},\:{d}_{xz})\:$orbitals of Y atoms (see also supplementary materials, Fig. S7). The Sc/Y-CrSiN is a magnetic monolayer with a magnetic moment of 1.09/1.19 *µ*_*B*_, which is more related to the difference between the outershell electrons of Cr and Sc/Y atoms. Additionally, the analysis of Löwdin charges shows that a partial charge of 0.78/0.45*e* is transferred from CrSiN to Sc/Y dopant.

Figure 4(c)/(d) shows the impact of Ti doping on the electronic structure of CrSiN, respectively. The semiconductive properties of Ti-CrSiN are preserved, with an indirect band gap of 0.20 eV. The electronic structures illustrate that the symmetry of spin-up and spin-down states leads to a zero magnetic moment. N and Cr contribute significantly to the DOS, while the dopants do not appear to contribute. The results of the electronic structures of Zr-CrSiN are similar to the corresponding spectrum of Ti-CrSiN, with a band gap of 0.25, such as near the Fermi energy (see supplementary materials, Fig. S8). Moreover, a partial charge of 0.92/0.21*e* moved from CrSiN to the Ti/Zr dopant.

The band structure and DOS/PDOS of V-CrSiN are shown in Fig. 4(e)/(f). Since V- and Nb-CrSiN have the same electronic structures, we do not display the results of Nb-CrSiN (see supplementary materials, Fig. S9). The top of the valence band and the entire conduction band are distorted due to the contribution of V states. The V states appear in the energy range from − 0.6 eV to 0.4 eV, and above 1.6 eV, the Fermi energy shifts towards the lower energy. The V/Nb-CrSiN monolayer is a non-magnetic metal. A partial charge of 0.85/0.15*e* transfers from CrSiN to V//Nb dopant.

Mo and Cr atoms are in the same group in the periodic table (6B); the electronic structures of Mo-CrSiN, Figs. 4(g)/(h), are similar to those of the corresponding electronic structures of pristine CrSiN, with a larger band gap of 0.52 eV. The contributions of Mo states appear at − 1 eV, 2.4 eV, and 2.8 eV. The conduction band is distorted as compared to the pristine structure due to the contribution of Mo states. Notably, the band gap of MoSi_2_N_4_ is 1.74 eV, and the band gap of substitutional Cr doping for MoSi_2_N_4_ is 1.2 eV^32^, indicating that the band gap value increases with increasing Mo doping concentration. The charge transfer from CrSiN to Mo is 0.95*e*.

**Fig. 6:**
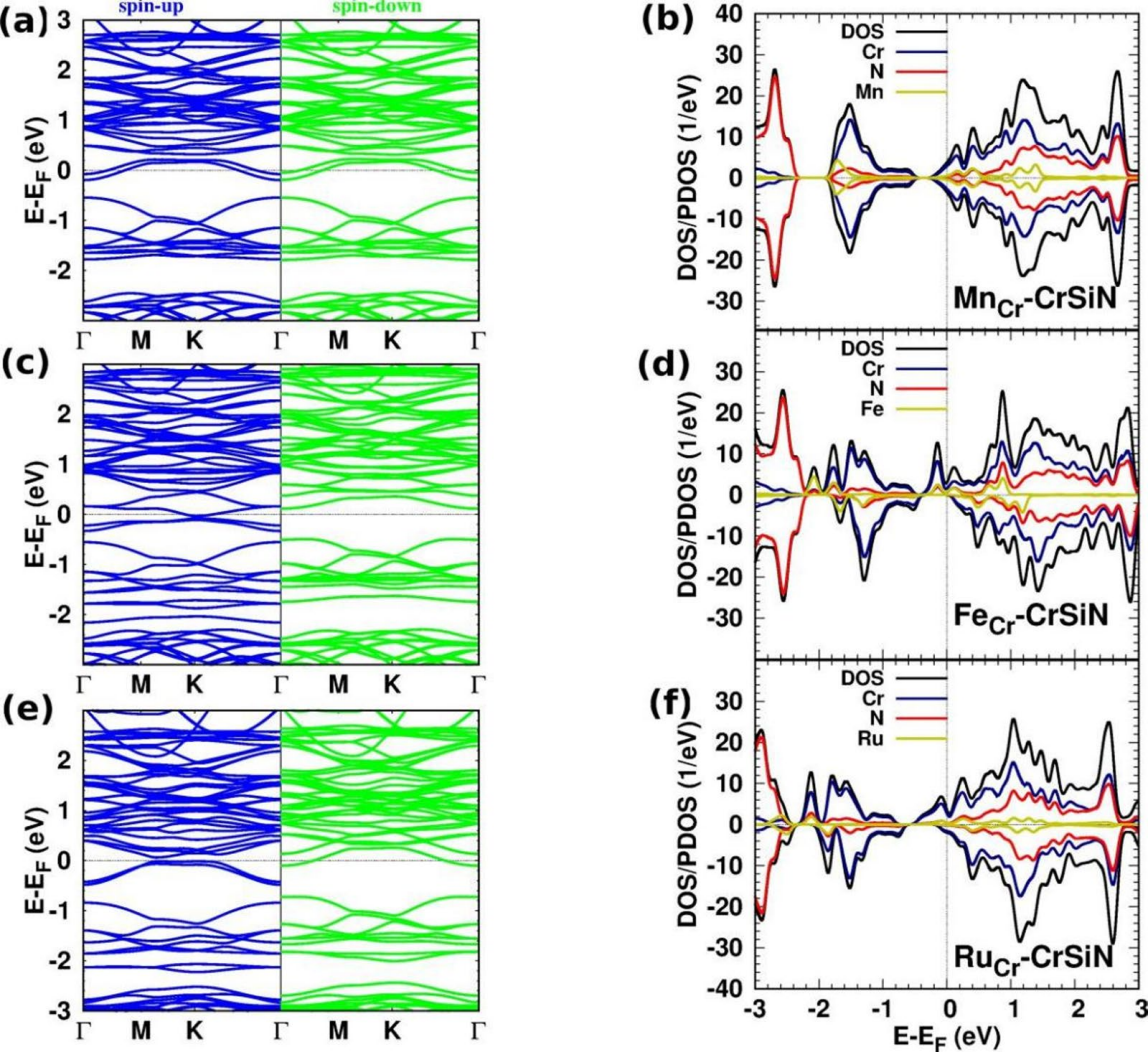
(The band structure, DOS/PDOS): (**a**,** b**) Mn-, (**c**,** d**) Tc-, (**e**,** f**) Fe-, and (**g**,** h**) Ru-CrSiN.

Figure 6(a)/(b) shows the electronic structure of Mn-CrSiN. Since the Mn atom has one more electron in its outer shell than the Cr atom, the Fermi energy shifts towards the conduction band, leading to the structure being n-type conducting. We only introduced Mn-CrSiN, not Tc-CrSiN, because there is no appreciable difference between them (see supplementary materials, Fig. S10). The contribution of Mn states is significantly larger compared to previous doping. The monolayers are nonmagnetic, and a partial charge of 0.81/0.20*e* transfers from Mn/Tc dopant to CrSiN sheet.

Figure 6(c)/(d) illustrates how the Fe dopant affects the CrSiN electronic structures. In comparison to pristine CrSiN, the entire electronic structure is distorted, ranging from − 2.1 eV to 3.0 eV. The asymmetry between the spin-up and spin-down states for electronic structures is apparent. The states fill the spin-up band gap, and the Fermi energy is shifted towards higher energy. The contribution of Fe states in the energy range from − 2.1 eV to 1.4 eV and dominates at − 2.1 eV (spin-up) and − 1.6 eV (spin-down). At the Fermi energy, the 3$\:{d}_{xz}$ of the Fe atom is dominated by a contribution from $\:{d}_{{x}^{2}-{y}^{2}}$ of Cr and Fe atoms (Fig. 5(b)). The Fe-CrSiN sheet is a half-metallic material, indicating that it can be utilized in spintronic devices. The magnetic moment of Fe-CrSiN is 1.84 *µ*_*B*,_ and a charge transfer is 1.99*e* from the host sheet to the Fe atom.

Regardless, the Ru atom is in the same group as Fe in the periodic table (group 8BI). Figure 6(e)/(f) shows a significant difference between Ru-CrSiN and Fe-CrSiN. The Ru-CrSiN is a metallic structure due to the position of the Fermi energy in the conduction band for both spin components. The contribution of Ru states is lower than that of Fe states (Fe-CrSiN), which means the effect of Ru doping is less than that of Fe doping. The Ru-CrSiN monolayer has a spin polarization ratio of 31%, a magnetic moment of 1.51 *µ*_*B*_, and a charge transfer of 0.91*e* from the Ru dopant to the CrSiN monolayer.

**Fig. 7:**
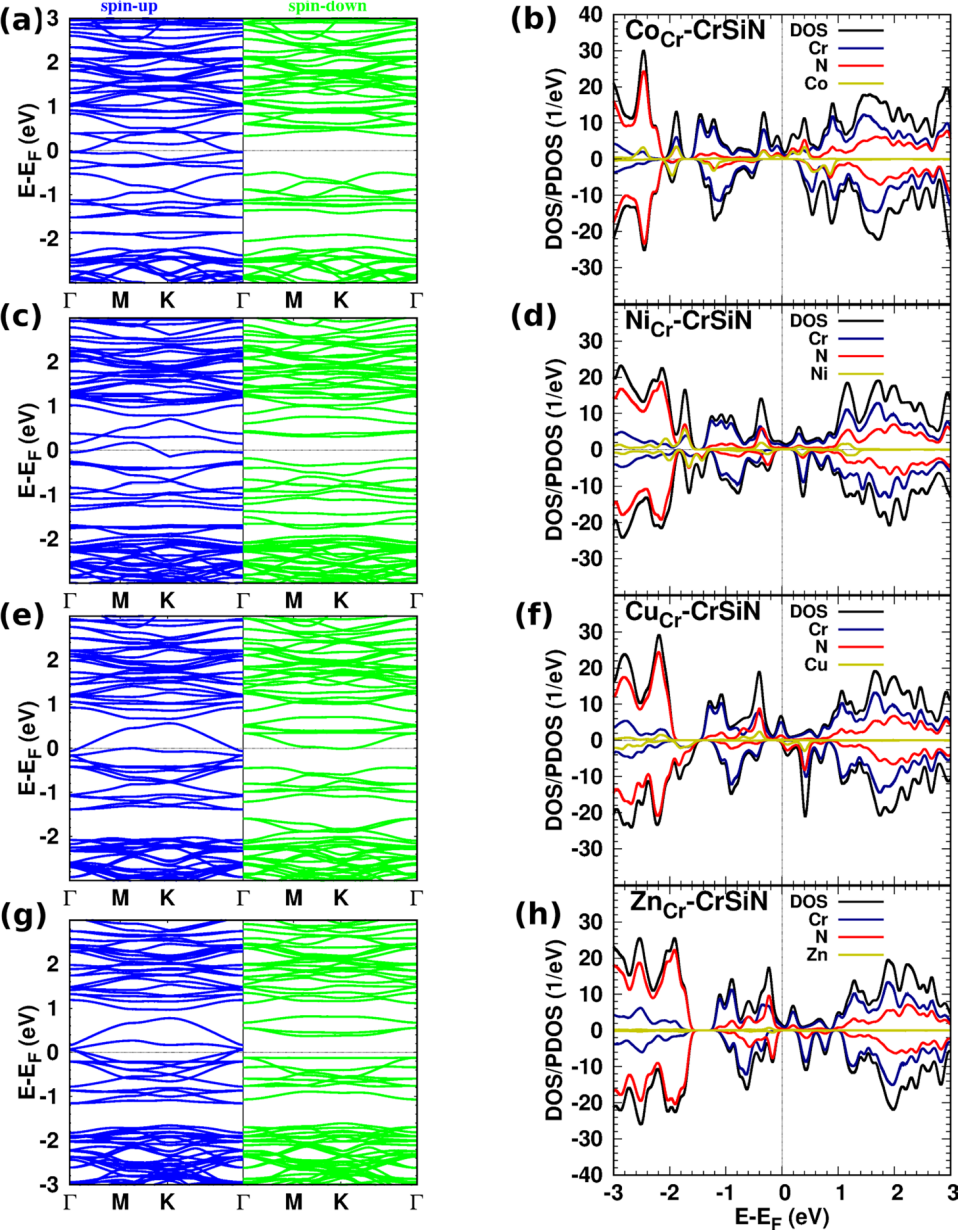
(The band structure, DOS/PDOS): (**a**,** b**) Co-, (**c**,** d**) Ni-, (**e**,** f**) Cu-, and (**g**,** h**) Zn-CrSiN.

When we replace one Cr by a Co atom in CrSiN, Fig. 7(a)/(b) shows that the electronic structures of Co-CrSiN are similar to the corresponding electronic structures of Fe-CrSiN. The 3$\:{d}_{xy}$ of Co atom is dominant and hybridizes with 3$\:{d}_{{x}^{2}-{y}^{2}}$ of Cr and Co atoms at the Fermi energy (Fig. 5(c)). For Rh-CrSiN, at the Fermi energy, the dominant 4$\:{d}_{xz}$ orbital hybridizes with 3($\:{d}_{xy},\:{d}_{xz})\:$orbitals of Cr atoms (see supplementary materials, Fig. S7). The structures indicate that Co- and Rh-CrSiN (see supplementary materials, Fig. S11) exhibit half-metallic properties, which means they are suitable for spintronic applications. Notably, the contribution of Rh states is less than that of Co states in the corresponding doping monolayers. The magnetic moment for Co/Rh-CrSiN is 2.91 *µ*_*B*_, which is more related to the difference in the number of electrons in the dopant atom and the replaced atom. A partial charge of 0.83/0.90*e* transfer into CrSiN from Co/Rh.

Figure 7 (c)/(d) displays the electronic structures of the Ni-CrSiN, and supplementary materials include Pd-CrSiN (Fig. S12). The Ni/Pd-CrSiN monolayer is a half-metallic sheet with a magnetic moment of 1.83/1.94 *µ*_*B*_ and charge transfer of 0.78/0.18*e* from Ni/Pd to CrSiN monolayer. The *d*-state contribution at the Fermi energy in Ni-CrSiN originates from the hybridization of 3$\:{d}_{{x}^{2}-{y}^{2}}$ of Cr and Ni atoms, and 3$\:{d}_{xy}\:$ of Ni atom (Fig. 5 (d)). In Pd-CrSiN, this contribution arises from the hybridization between 3$\:{d}_{{x}^{2}-{y}^{2}}$ of Cr and 4$\:{d}_{{x}^{2}-{y}^{2}}$ of Pd (see supplementary materials, Fig. S7). Due to the nearly closed *d* orbital shell of Ni/Pd atom, their *d*-state contributions are smaller than those in Sc/Y-, Fe-, and Co/Rh-CrSiN.

Regarding Cu-CrSiN, the electronic structures are depicted in Fig. 7(e)/(f) (Ag dopant is presented in supplementary materials, Fig. S13). Both doped monolayers are metallic. The contribution of Cu/Ag states is small compared to Cr and N states around the Fermi energy, and the contribution of Ag states is smaller than the contribution of Cu states. Both doped monolayers are magnetic, with a magnetic moment of 4.55/4.54 for Cu/Ag-CrSiN, which is approximately related to the difference in the number of outer-shell electrons in Cu/Ag and Cr atoms. Due to the difference in the DOS values of spin-up and spin-down states, Cu/Ag-CrSiN exhibits a SPR of 71%/42%, making it suitable for spin-filter devices. The charge transfer from the sheet to Cu/Ag is 0.08/0.09*e*.

The last doped structures in this study are Zn-CrSiN and Cd-CrSiN monolayers. Due to the similarity between the two doped structures, we present the electronic structures of Zn-CrSiN in Fig. 7(g)/(h), while the corresponding spectra for Cd-CrSiN can be found in the supplementary materials (Fig. S14). The Zn/Cd-CrSiN has an SPR of 100%, which is beneficial for spintronic devices. The contribution of Zn/Cd states is very small in the shown energy range. The Zn/Cd atom is closed *d* outershell, the contribution of *d* states originates from the hybridization of 3($\:{d}_{{x}^{2}-{y}^{2}},{d}_{xy})$ orbitals in Cr atoms (Fig. 5(e) and Fig. S7). The asymmetry of spin-up and spin-down DOSs shows the structure is magnetic with a value of 1.93/2.37 *µ*_*B*_, and the charge transfer from Zn/Cd dopant to the CrSiN monolayer is 0.10/0.19*e*.

**Fig. 8:**
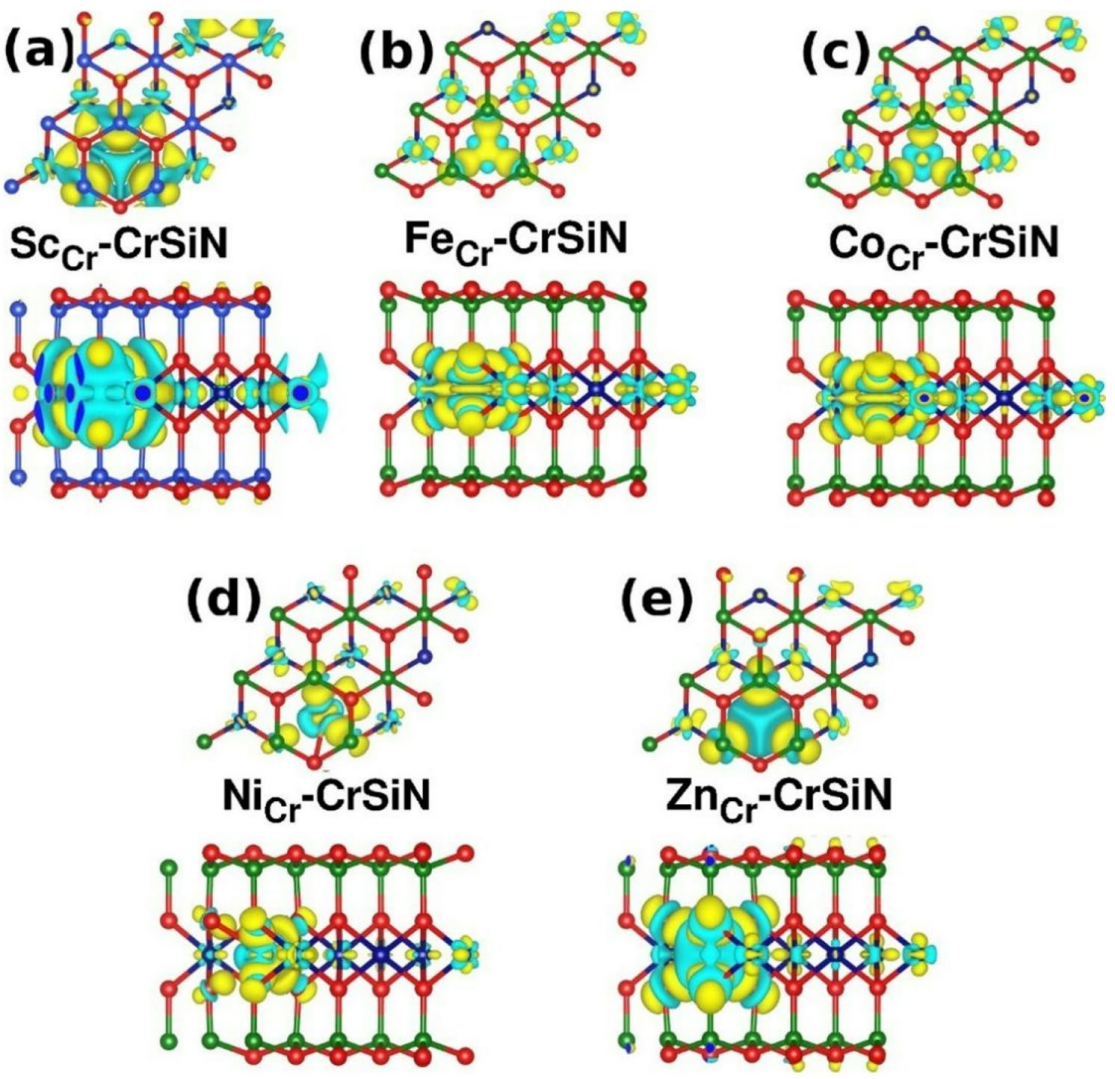
Charge density difference of (**a**) Sc-, (**b**) Fe-, (**c**) Co-, (**d**) Ni-, and (**e**) Zn-CrSiN. Top and side views are shown for each configuration. Yellow and cyan regions indicate the electron accumulation and depletion.

The charge density difference for doped CrSiN with Sc, Fe, Co, Ni, and Zn atoms is displayed in Fig. 8 (Fig. S15 in the supplementary includes the charge density difference distributions of Y-, Rh-, Pd-, and Cd-CrSiN monolayers). The figures show how substitutional doping at the Cr site changes the distribution of charge. We observe a high charge density around the dopant atoms, indicating a charge transfer from Sc/Y, Ni, and Zn/Cd dopants (depletion) to the sheet, while a charge transfer occurs from the sheet to the Fe, Co/Rh, and Pd dopants (accumulation). The spatial distribution of charge accumulation surrounding the dopant sites indicates improved covalent bonding.

## Conclusion

We employed density functional theory to investigate the structural, electronic, and magnetic properties of pristine and doped CrSi_2_N_4_ (CrSiN) single layers with substitutional 3d/4d transition metal doping (TM) at the Cr site. The bond lengths of TM-Cr/N increase with the atomic size of TM, and the formation/binding energy can be explained by the atomic size and electronegativity of TM relative to those of Cr. The calculated work functions for TM-CrSiN range from 4.60 eV to 5.54 eV for TM = 3 d and from 4.38 eV to 5.50 eV for TM = 4d. The work function behavior is related to the electronegativity of TM, where an increase in TM electronegativity results in a decrease in the work function. The doped structures with Ti/Zr, V/Nb, Mo, and Mn/Tc are nonmagnetic monolayers. In contrast, the other doped monolayers are magnetic monolayers with different magnetic moments depending on the difference in the number of electrons in the valence shell between the dopant (TM) and the Cr atom. When Ti-, Zr-, and Mo-CrSiN monolayers are semiconductors with a band gap of 0.20 eV, 0.25 eV, and 0.52 eV, respectively. The structures with Sc/Y, Fe, Co/Rh, Ni/Pd, and Zn/Cd dopants are half-metallic, which makes them suitable monolayers for spintronic applications. We find Cu- and Ag-CrSiN sheets are metal structures and can be utilized in spin-filter devices.

## Supplementary Information

Below is the link to the electronic supplementary material.

Supplementary Material 1

## Data Availability

The datasets used and/or analyzed during the current study are available from the corresponding author on reasonable request.
