# Supplementary material for: The structural, electronic, and magnetic properties of substitutional transition metal doping in CrSi2N4 monolayer
Source: Sci Rep. 2025 Dec 18;15:44134. doi: 10.1038/s41598-025-30838-0 (PMC12717429; doi:10.1038/s41598-025-30838-0)
Supplement: Supplementary file 1 — Supplementary Material 1 [file 41598_2025_30838_MOESM1_ESM.docx]

**The structural, electronic, and magnetic properties of**

**substitutional transition metal doping in CrSi_2_N_4_ monolayers**

Mohamed A. Abdelati^2^ and Mohamed M. Fadlallah^1^

^1^Department of Physics, Faculty of Science, Benha University, 13518 Benha, Egypt

^2^National Institute of Laser Enhanced Sciences, Cairo University, ElGiza, Egypt


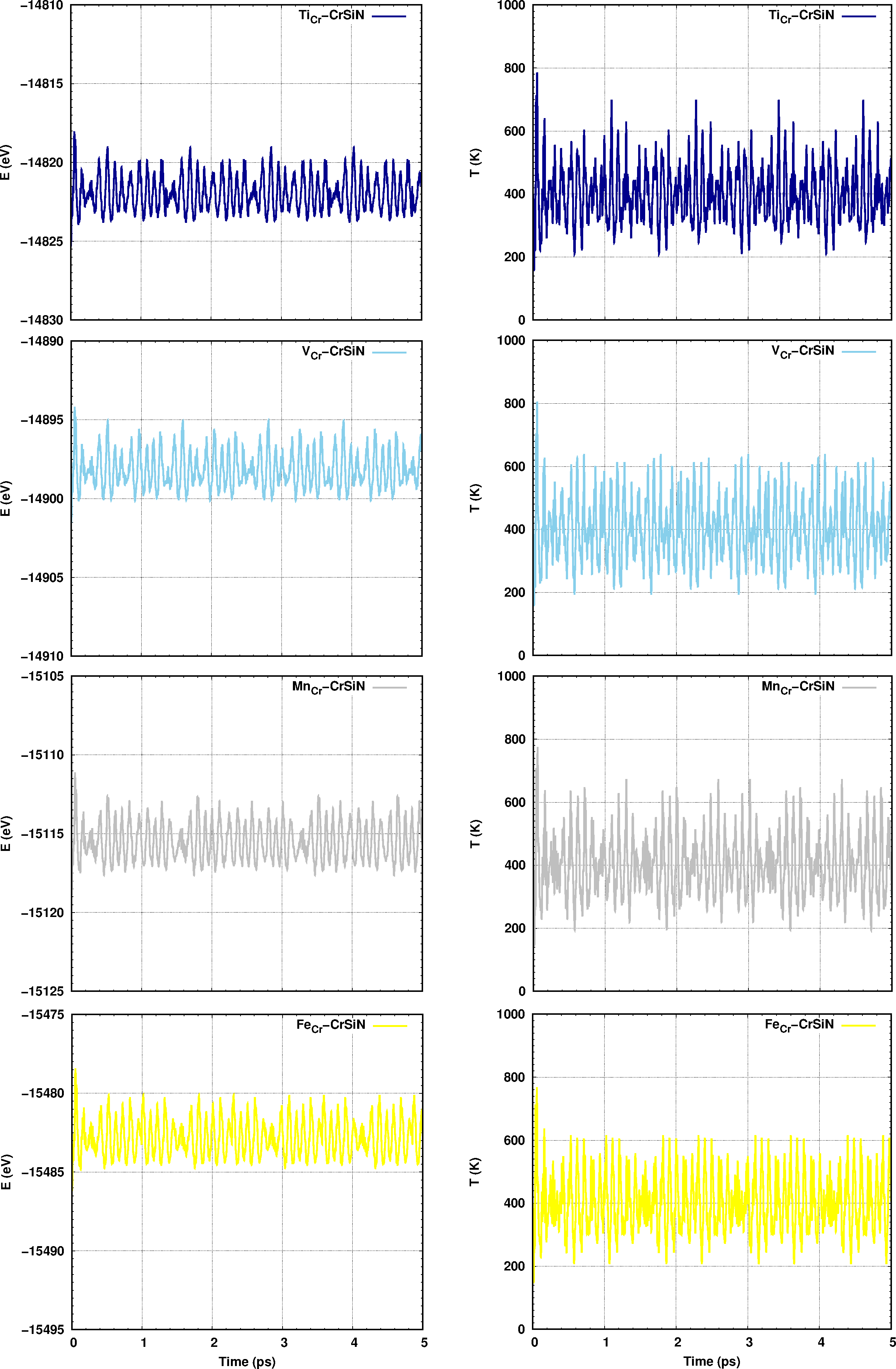


Figure S1: AIMD simulations of Ti-, V-, Mn- and Fe-CrSiN at 400 K


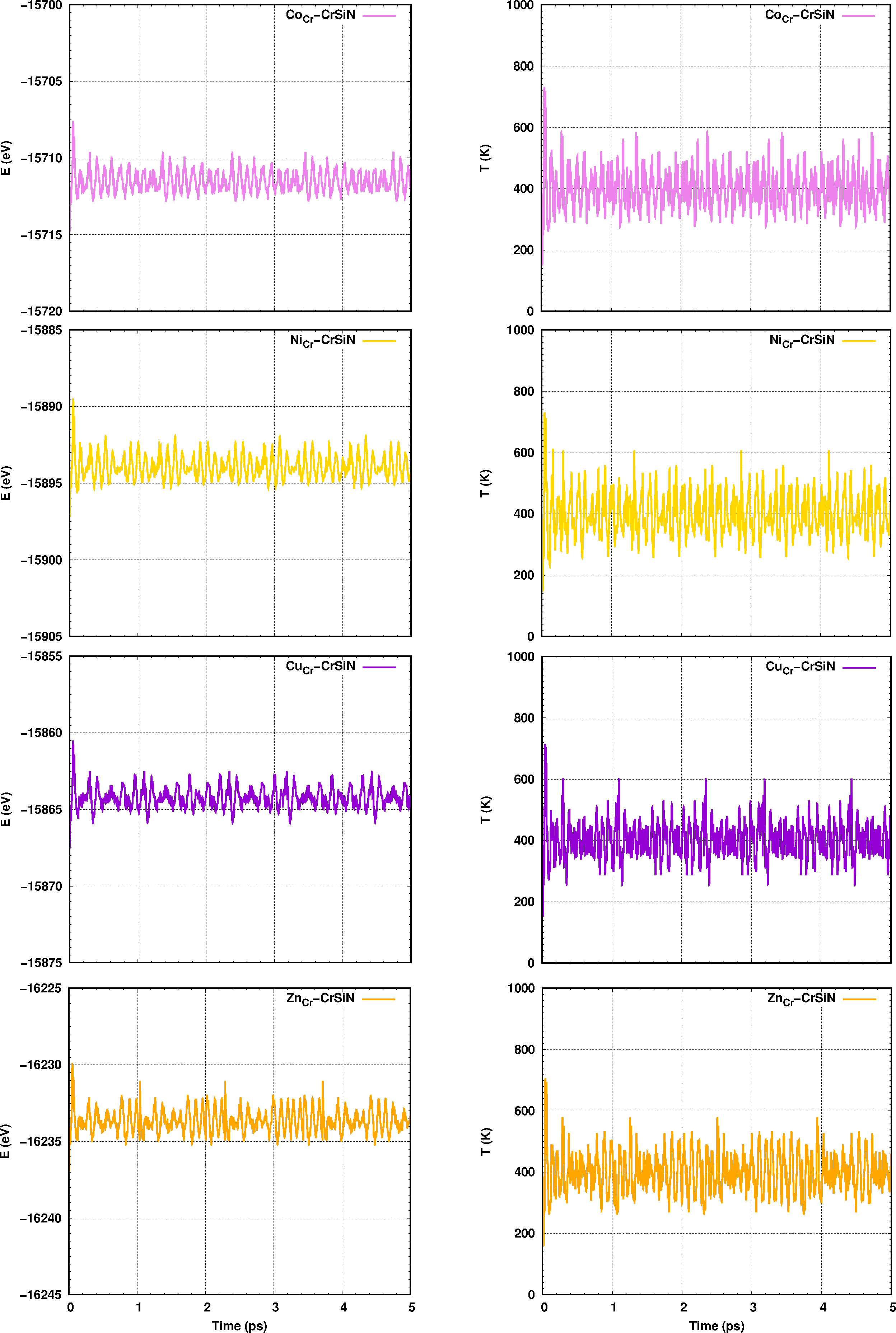


Figure S2: AIMD simulations of Co-, Ni-, Cu- and Zn-CrSiN at 400 K


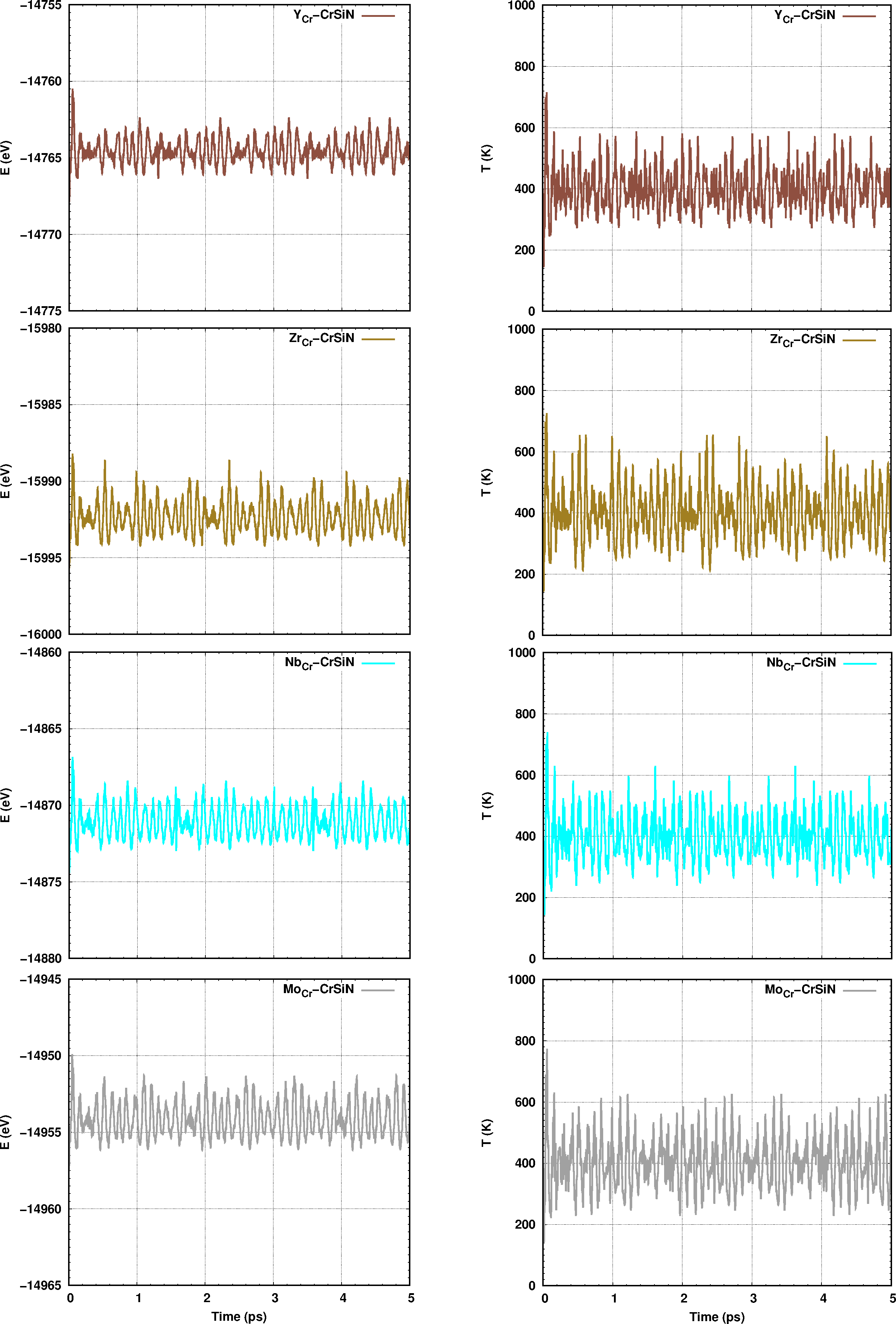


Figure S3: AIMD simulations of Y-, Zr-, Nb- and Mo-CrSiN at 400 K


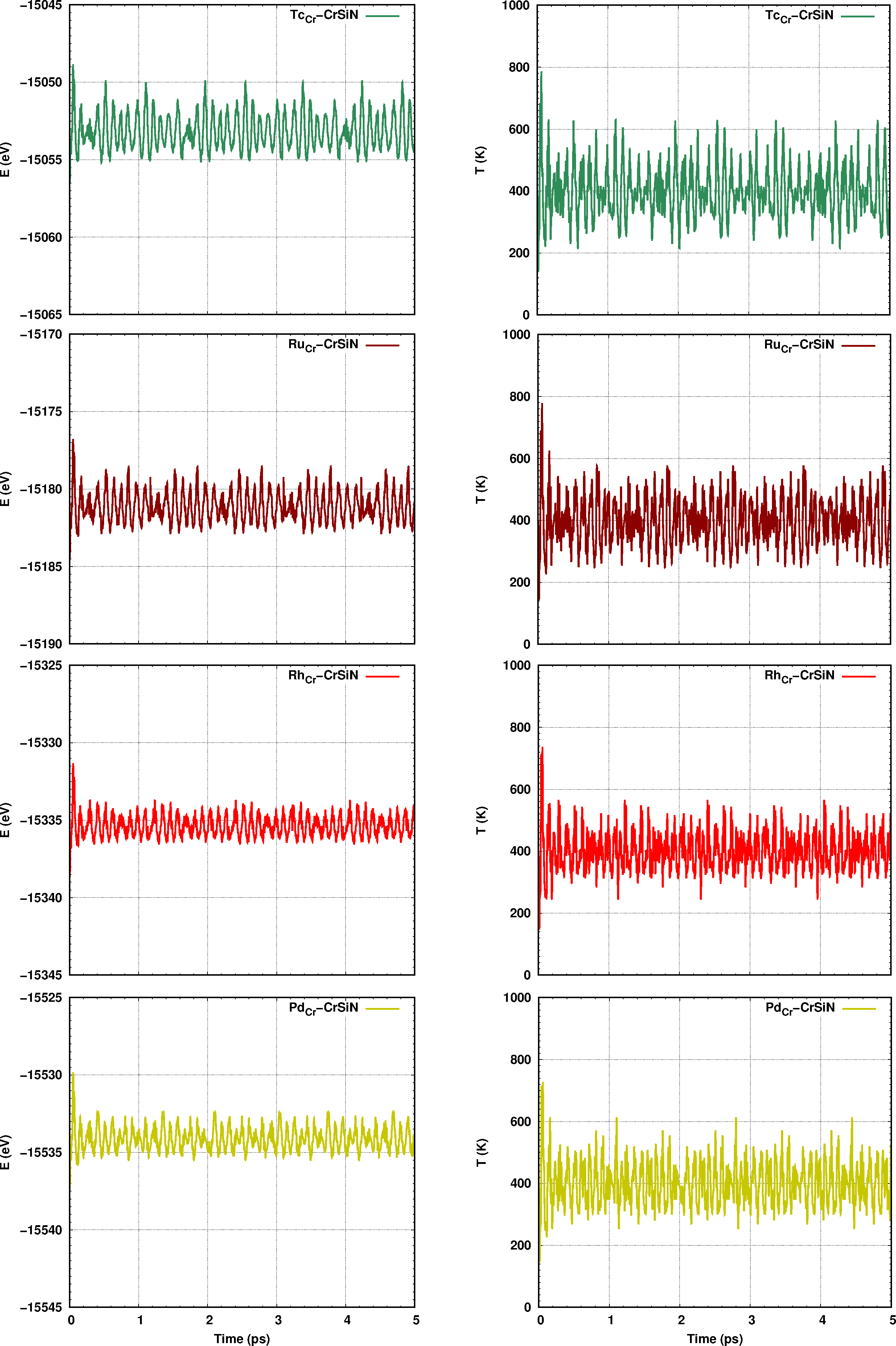


Figure S4: AIMD simulations of Tc-, Ru-, Rh- and Pd-CrSiN at 400 K


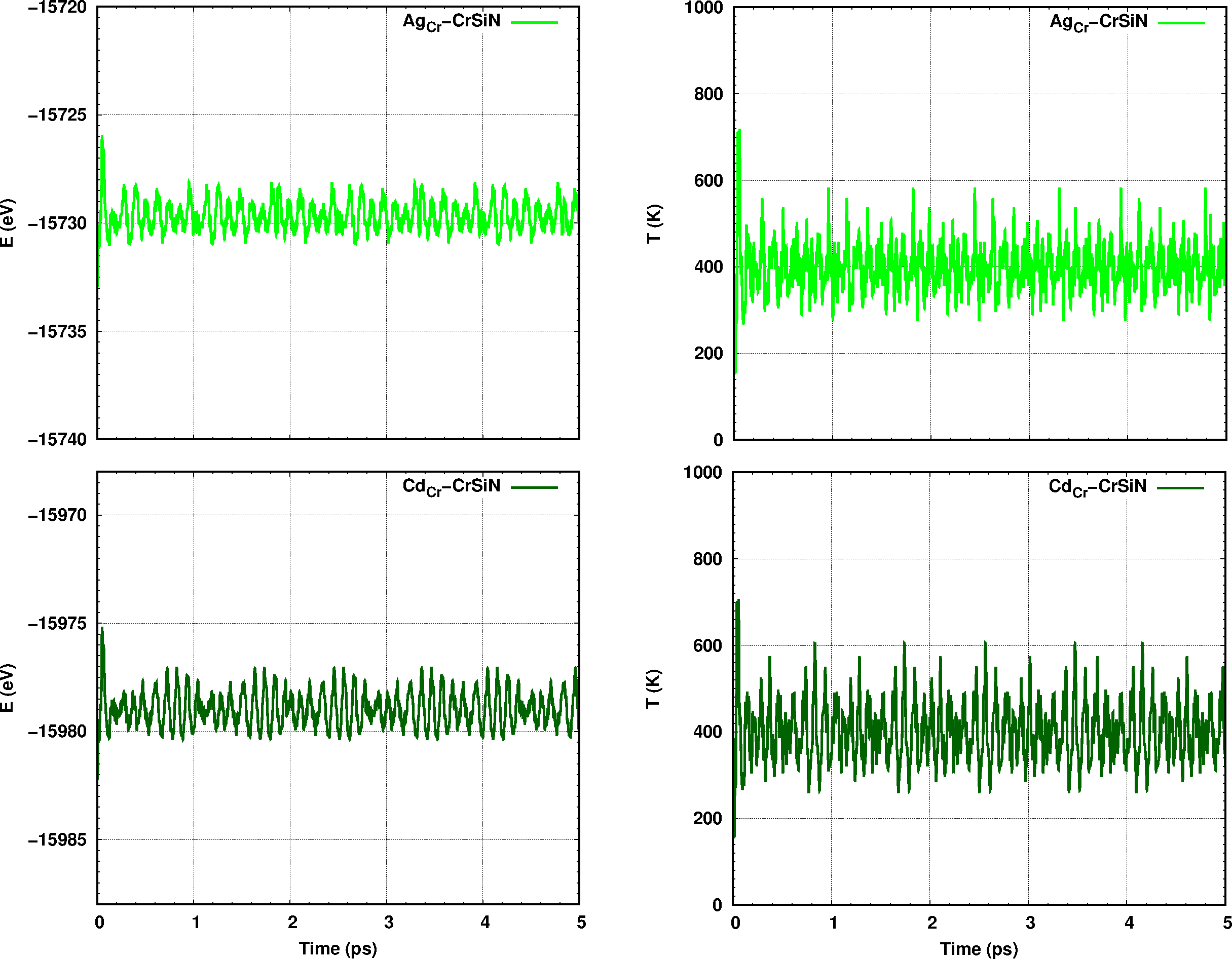


Figure S5: AIMD simulations of Ag- and Cd-CrSiN at 400 K


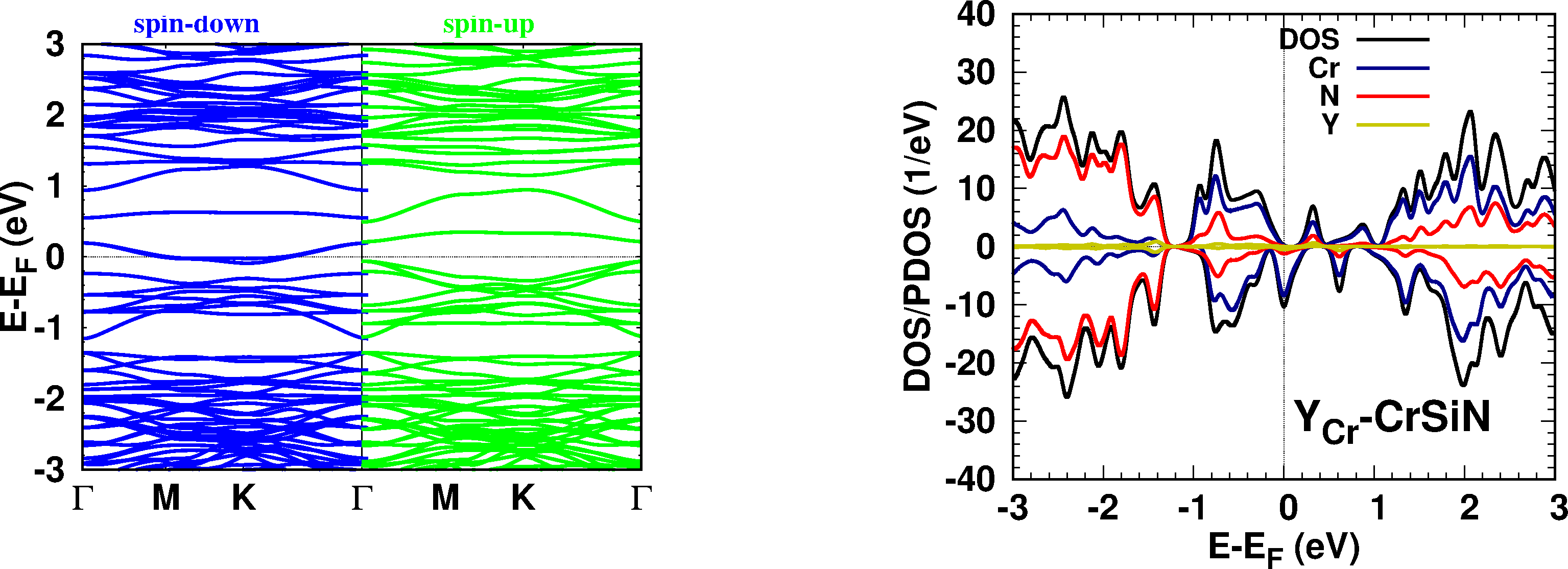


Figure S6: The band structure of Y-CrSiN and the corresponding DOS/PDOS

.


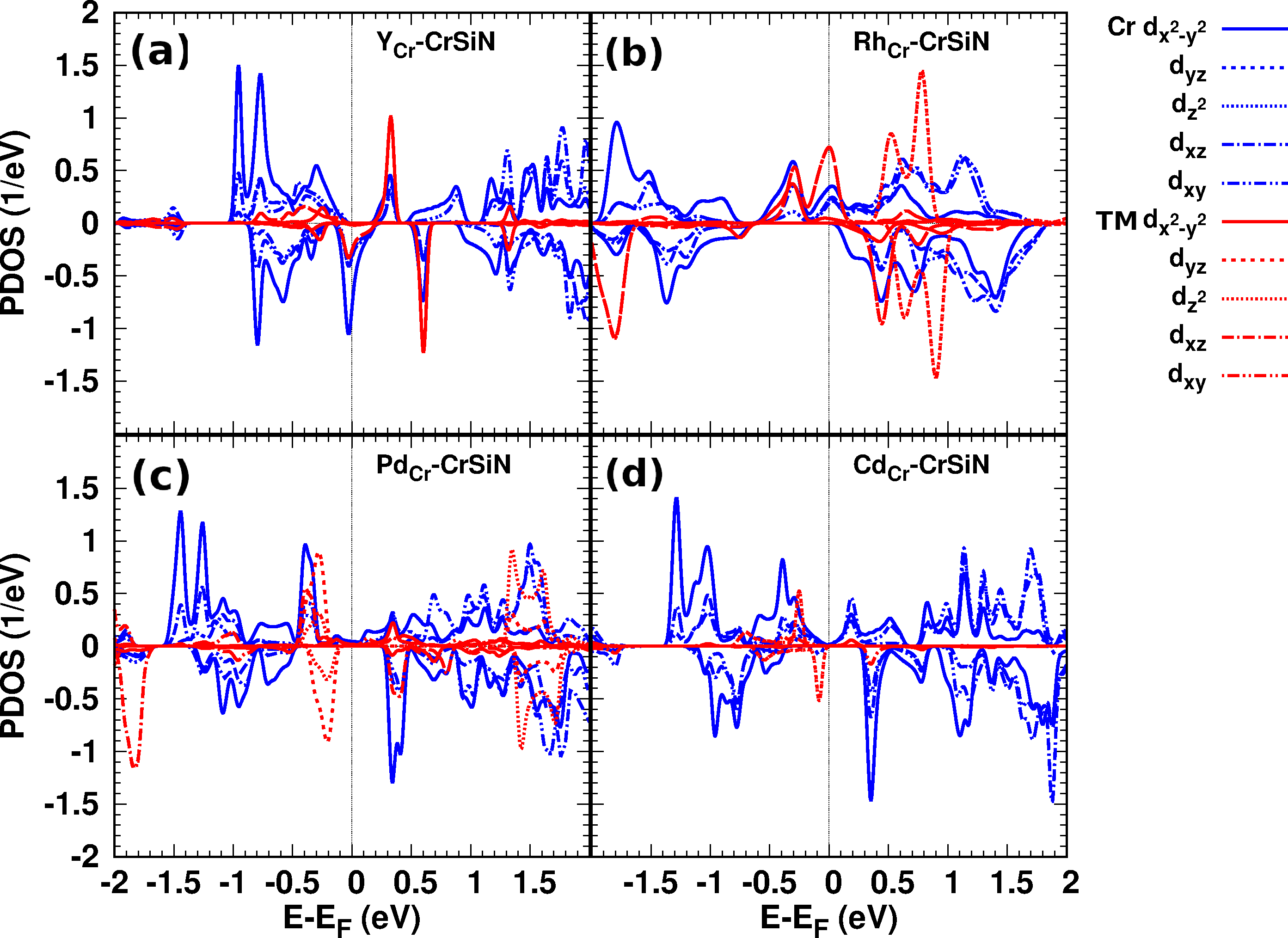


FIG S7: The PDOS for (3d) orbitals of Cr and TM: (a) Y-, (b) Rh-, (c) Pd-, and (d) Cd-CrSiN. The TM refers to transition metals.


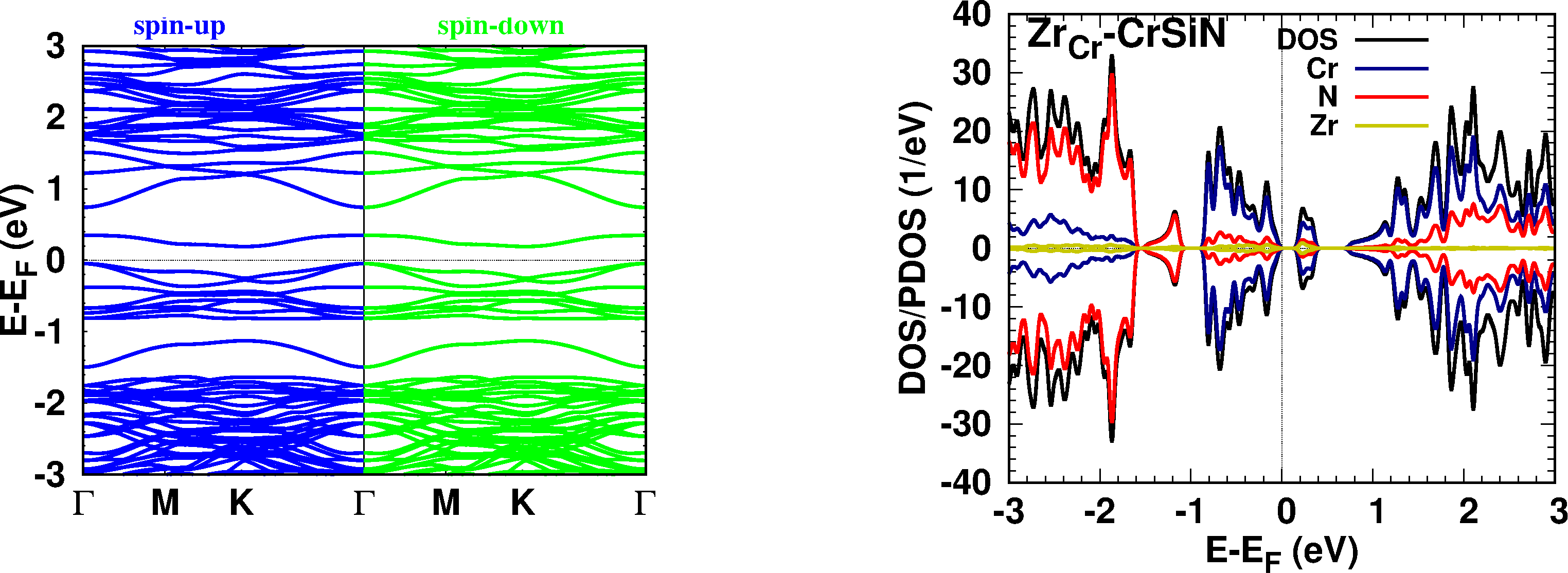


Figure S8: The band structure of Zr-CrSiN and the corresponding DOS/PDOS


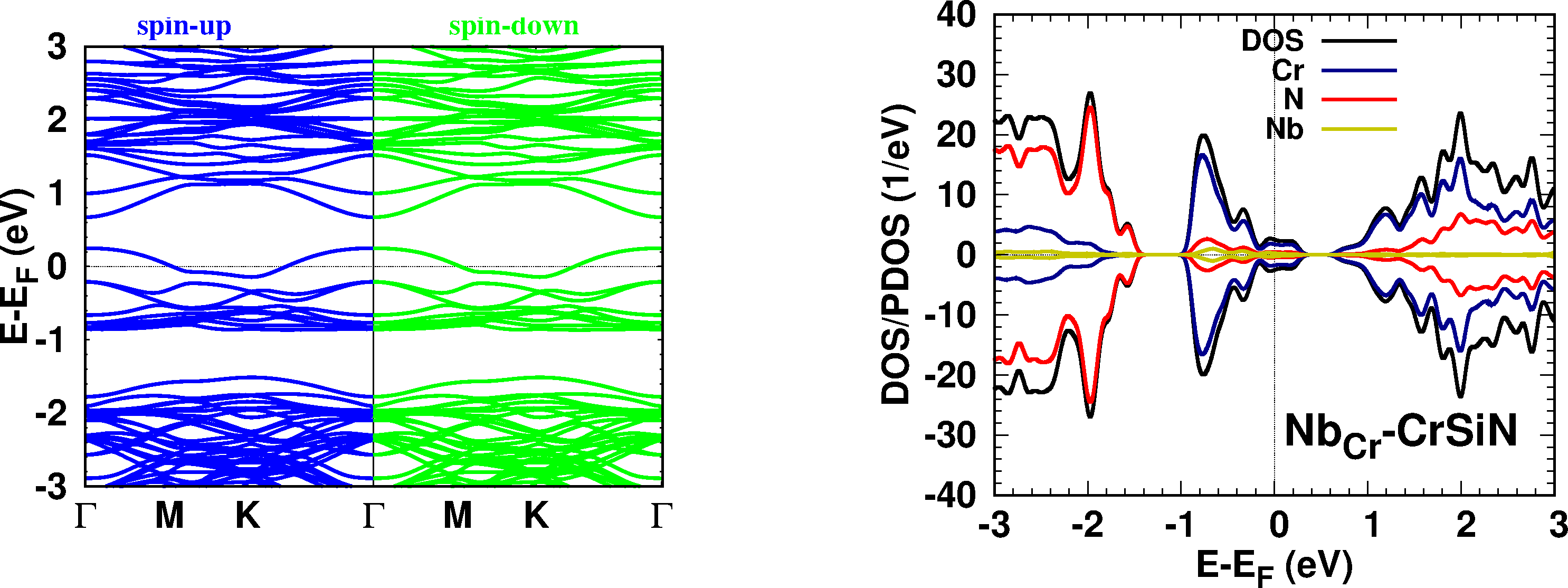


Figure S9: The band structure of Nb-CrSiN and the corresponding DOS/PDOS


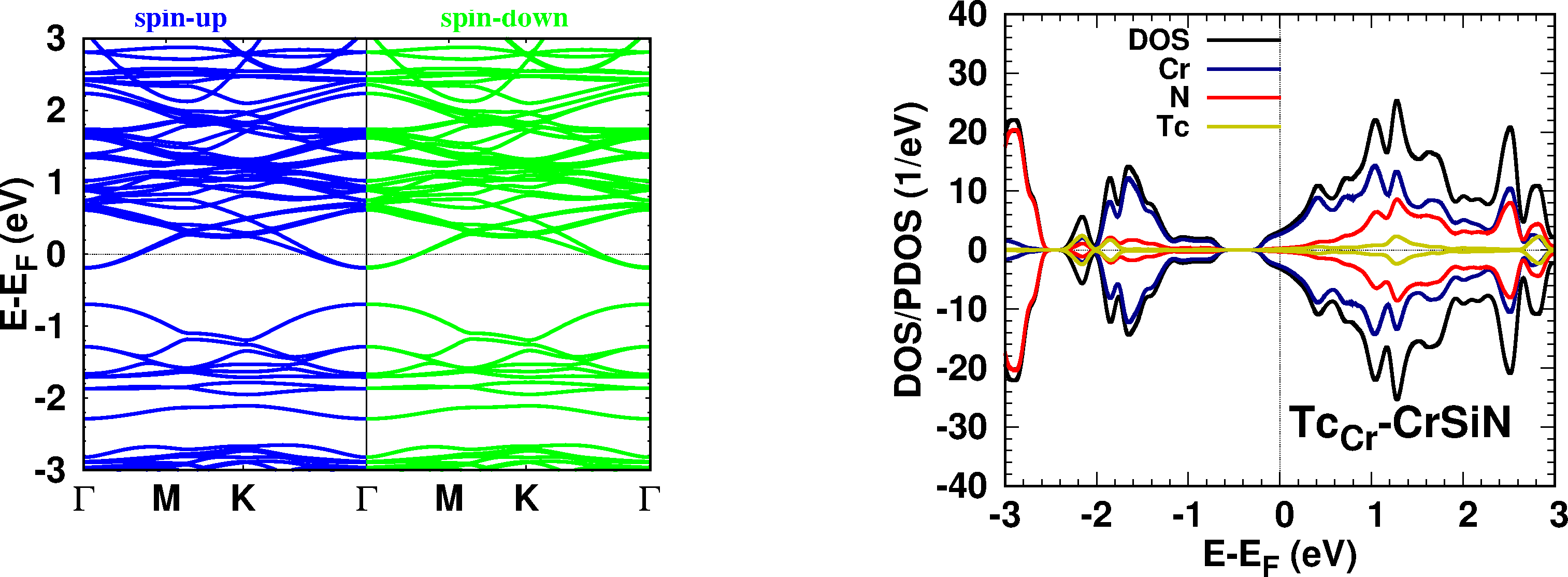


Figure S10: The band structure of Cd-CrSiN and the corresponding DOS/PDOS


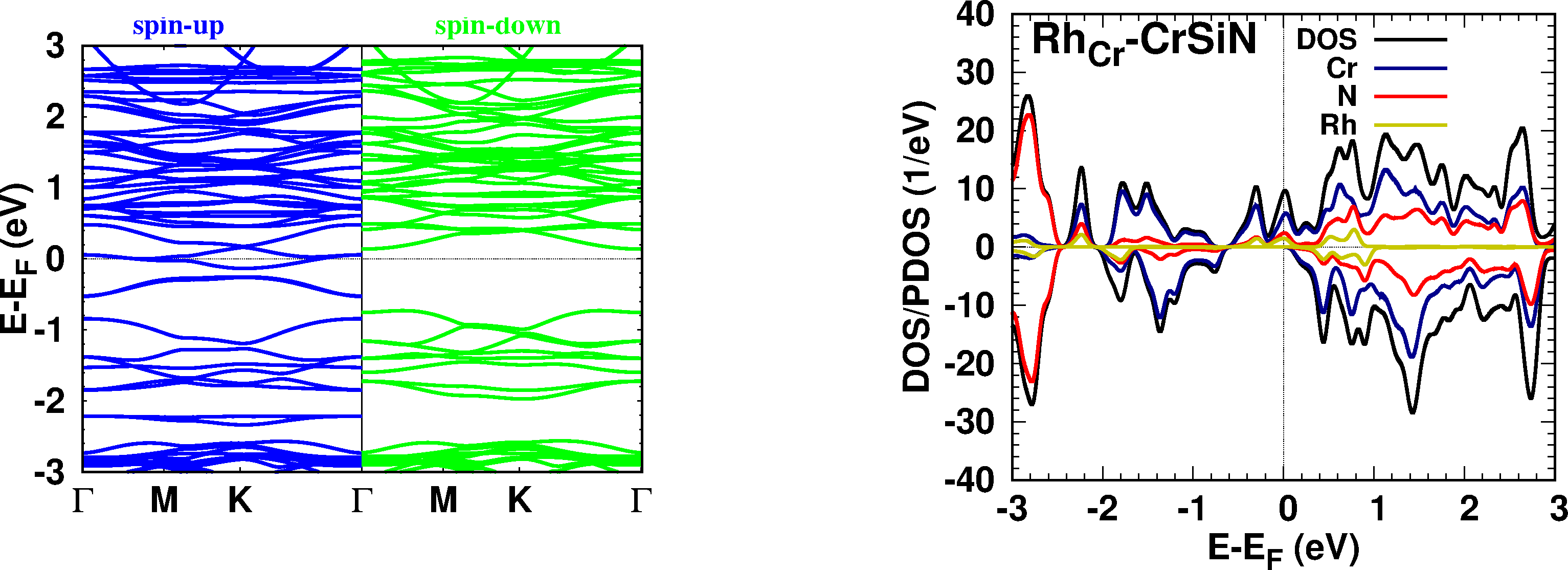


Figure S11: The band structure of Rh-CrSiN and the corresponding DOS/PDOS


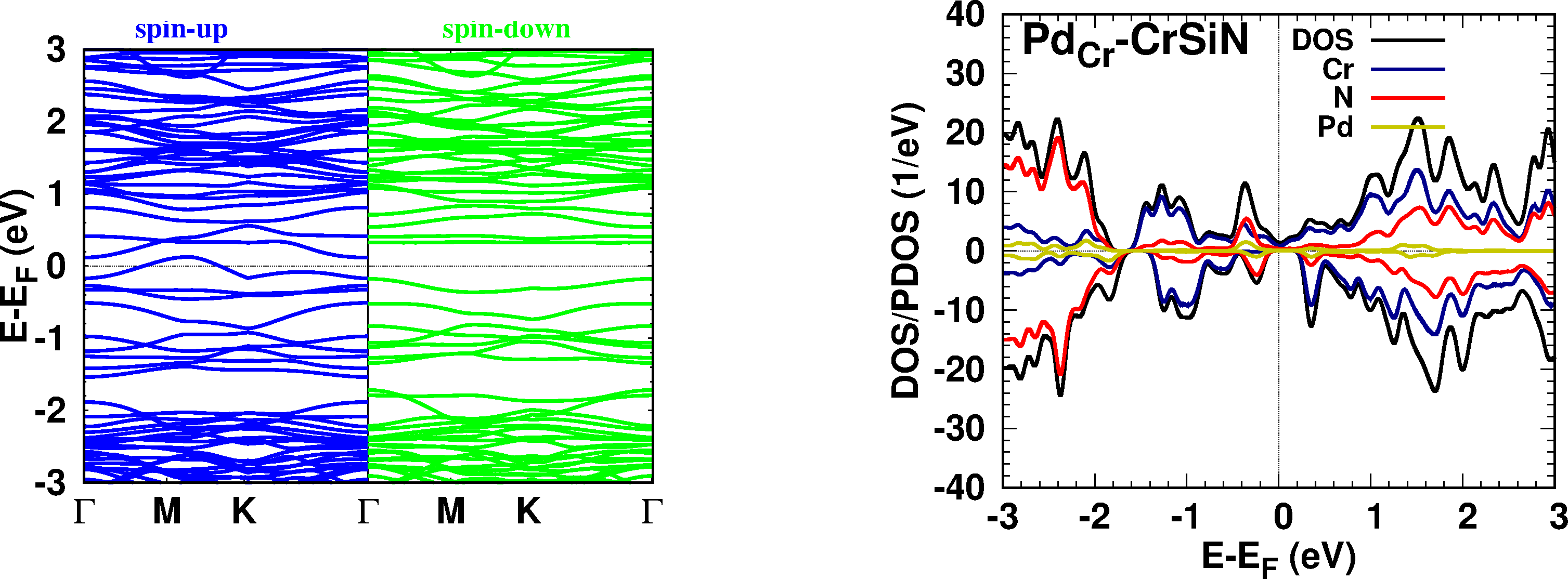


Figure S12: The band structure of Pd-CrSiN and the corresponding DOS/PDOS


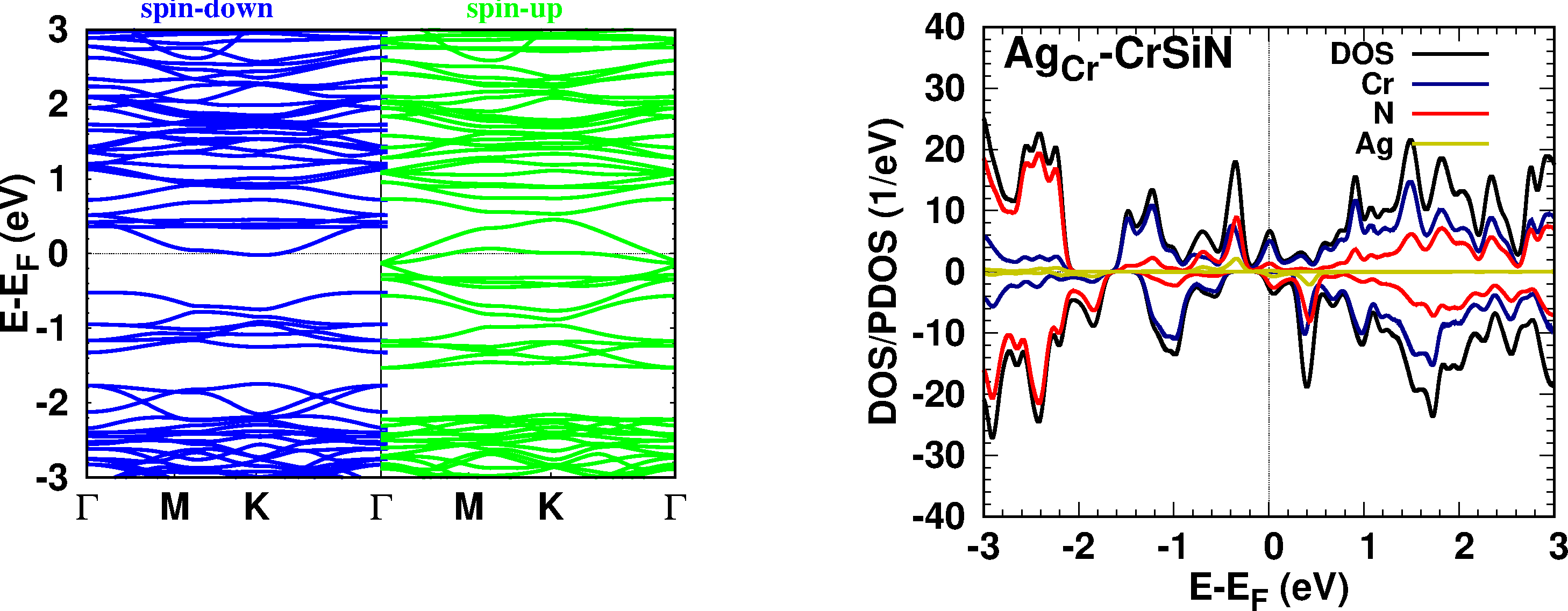


Figure S13: The band structure of Ag-CrSiN and the corresponding DOS/PDOS


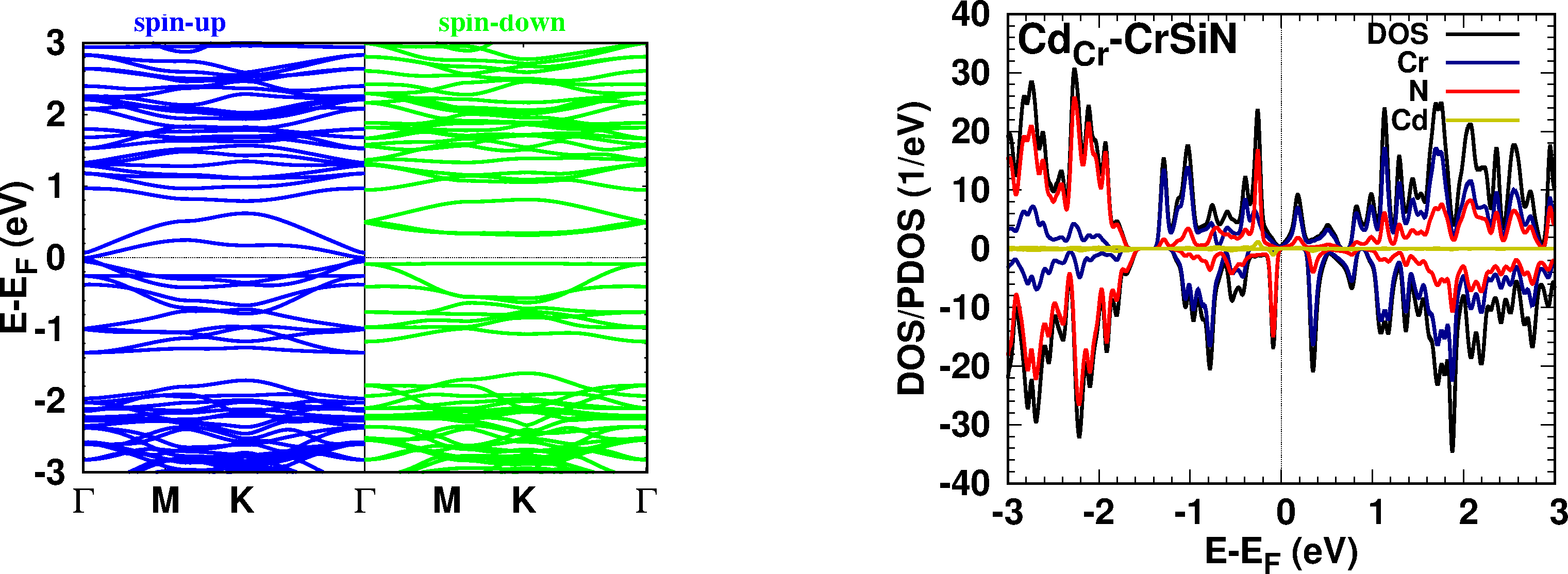


Figure S14: The band structure of Cd-CrSiN and the corresponding DOS/PDOS


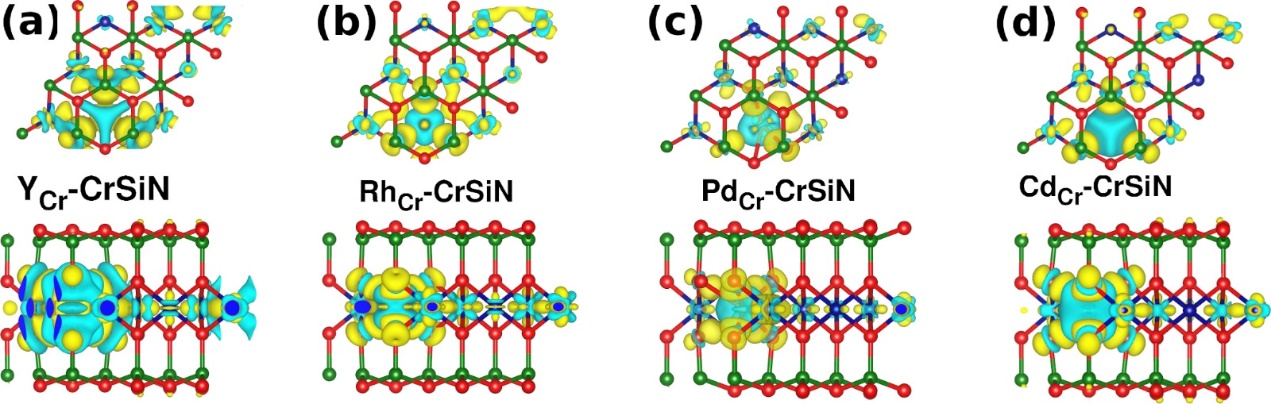


Figure S15: Charge density difference of (a) Y-, (b) Rh-, (c) Pd-, and (d) Cd-CrSiN. Top and side views are shown for each configuration. Yellow and cyan regions indicate the electron accumulation and depletion.
